# Pentahydroxy flavonoid isolated from *Madhuca indica* ameliorated adjuvant-induced arthritis via modulation of inflammatory pathways

**DOI:** 10.1038/s41598-021-97474-2

**Published:** 2021-09-09

**Authors:** Yongliang Tang, Daotao Xie, Wenqing Gong, Hongtao Wu, Yi Qiang

**Affiliations:** 1 Department of Orthopaedics, Xi’an Jiaotong University, Xi’an, 710000 China; 2 Department of Orthopaedics, Xi’an Central Hospital, Xi’an, 710000 China; 3 Department of Technology, Shaanxi Nuoxing Youchuang Medical Research Center, Xi’an, 710032 China; 4 Department of Ultrasound, Xijing Hospital, the Fourth Military Medical University, Xi’an, 710032 China; 5 Department of Gynecology, Xianyang Central Hospital, Xianyang, 712000 China; 6 Department of Orthopaedics, The Affiliated Hospital of Northwest University, Xi’an No. 3 Hospital, Xi’an, 710018 China

**Keywords:** Pharmacology, Rheumatoid arthritis, Inflammatory diseases, Drug discovery, Rheumatology

## Abstract

Rheumatoid arthritis (RA) is an autoimmune disease associated with advanced joint dysfunction. *Madhuca indica* J. F. Gmel, from the family Sapotaceae, is an Indian medicinal plant reported to have an array of pharmacological properties. The aim of present investigation was to determine the anti-arthritic potential of an isolated phytoconstituent from methanolic leaf extract of *Madhuca indica* (MI-ALC) against FCA-induced experimental arthritis. Polyarthritis was induced in female rats (strain: Wistar) via an intradermal injection of FCA (0.1 mL) into the tail. Polyarthritis developed after 32 days of FCA administration. Then rats were treated orally with an isolated phytoconstituent from MI-ALC at doses of 5, 10, and 20 mg/kg. Findings suggested that High-Performance Thin-Layer Chromatography, Fourier-Transform Infrared Spectroscopy, and Liquid Chromatography-Mass Spectrometry spectral analyses of the phytoconstituent isolated from MI-ALC confirmed the structure as 3,5,7,3′,4′-Pentahydroxy flavone (i.e., QTN). Treatment with QTN (10 and 20 mg/kg) showed significant (*p* < 0.05) inhibition of increased joint diameter, paw volume, paw withdrawal threshold, and latency. The elevated synovial oxidative stress (Superoxide dismutase, reduced glutathione, and malondialdehyde) and protein levels of Tumor necrosis factor-α (TNF-α) and Interleukin (ILs) were markedly (*p* < 0.05) reduced by QTN. It also effectively (*p* < 0.05) ameliorated cyclooxygenase-2 (COX-2), Nuclear factor of kappa light polypeptide gene enhancer in B cells (NF-kβ) and its inhibitor-α (Ikβα), and ATP-activated P2 purinergic receptors (P2X7) protein expressions as determined by western blot analysis. In conclusion, QTN ameliorates FCA-induced hyperalgesia through modulation of elevated inflammatory release (NF-kβ, Ikβα, P2X7, and COX-2), oxido-nitrosative stress, and pro-inflammatory cytokines (ILs and TNF-α) in experimental rats.

## Introduction

Rheumatoid arthritis (RA) is a complex, autoimmune, and inflammatory disorder associated with joint and synovial membrane inflammation, deformity, cartilage destruction, and pain, leading to loss of joint function and restricted limb motions^1,2^. RA shows symmetrical progression affecting metacarpophalangeal and metatarsophalangeal joints, proximal interphalangeal, ankle, and wrist^3^. This immune-inflammatory arthritis affects about 1–2%, i.e., nearly 20 million people worldwide, and the incidence is three times higher in women between the age of 40 and 50 years than men^4,5^. It is associated with significantly higher lifetime costs along with a poor quality of life.

The use of NSAIDs (Non-steroidal anti-inflammatory drugs (including ibuprofen and aceclofenac), DMARDs (disease-modifying anti-rheumatic drugs including methotrexate and Cyclosporin A), anti-CD20 and anti-TNFα (anti-tumor necrosis factor) (such as adalimumab, infliximab) are currently available therapies for management of RA^2^. These treatments halt the progression of disease via suppression of immunological response. However, such treatment regimens provided symptomatic relief in a few patients and showed several adverse events, including gastrointestinal discomfort, increased cardiovascular risk, and nephrotoxicity^2^. Thus, there is a need for conventional and alternative therapy with significant safety and efficacy. Numerous researchers have extensively utilized various animal models of Adjuvant induced arthritis (AIA) to analyze the efficiency of herbal moieties. Polyarthritis induced by Freund’s complete adjuvant (FCA) is one of the most employed AIA animal models of arthritis that possess many common features with human rheumatoid arthritis^1,2,6,7^.

*Madhuca indica* J. F. Gmel (family Sapotaceae) is an Indian medicinal plant found in moist habitats and has been reported to have various pharmacological properties, such as anti-inflammatory, hepatoprotective, antiulcer, analgesic, antidiabetic, cardioprotective, and wound healing^8–14^. Literature survey revealed that *Madhuca indica* contains chemical constituents such as quercitrin, quercetin, myricitrin, myricetin, erythritol, β-carotene xanthophylls, n-octacosanol, n-hexacosanol, palmitic acid, oleanolic acid, etc.^15^. A recent study on Pentahydroxy flavone, an isolated phytoconstituent from *Madhuca indica* methanol extract*,* showed antiulcer activity via inhibition of cyclooxygenase (COX)-II, ILs, TNF-α, and iNOs^13^. Researchers have reported that Pentahydroxy flavone present in *Madhuca indica* modulated expression of peroxisome proliferator-activated receptor-gamma (PPAR-γ) and nuclear factor-like 2 (Nrf2) as well as attenuated elevated oxidative stress to exert its cardioprotective activity^14^. Traditionally, *Madhuca indica* has been prescribed for management of arthritis and associated pain^16,17^. Additionally, a study reported the anti-inflammatory potential of *Madhuca longifolia* ethanolic extract in experimental rats^18^. Our preliminary study (unpublished) suggested that *Madhuca indica* leaves methanolic extract exhibits potent anti-arthritic activity. However, to the best of our knowledge, the potential phytoconstituents responsible for its anti-arthritic activity remain to be determined. Given this premise, the present investigation was undertaken with the objective of investigating the anti-arthritic activity of a phytoconstituent isolated from the leaves of *Madhuca indica* methanolic extract against FCA-induced experimental arthritis.

## Material and methods

### Animals

Wistar rats (Adult female, 150–180 g) were procured from the animal house of Xi’an Central Hospital. The rats were maintained in-house at 24 ± 1 °C, 12 h:12 h dark–light cycle, with standard pellet feed and filtered water during the experimental time of 0800 h and 1700 h in quiet laboratory environment. The Xi’an Central Hospital’s Institutional animal ethics committee approved all the experimental research protocols (no. XCH202013771).

### Chemicals and reagents

Reagents used during the study included rat specific ELISA (enzyme-linked immunosorbent assay) kit for ILs (IL-1β, and IL-6) and TNF-α (Bethyl Laboratories Inc., Montgomery, TX, USA), primary antibodies of NF-kβ, Ikβα, COX-2, P2X7, and GAPDH (Abcam, Cambridge, MA, USA), FCA (Sigma Aldrich, St. Louis, USA), microliter syringe (Hamilton, Bonaduz, Switzerland), ethyl acetate, methanol, formic acid, toluene (Merck Life Science Pvt Ltd, India), and high-performance thin-layer chromatography (HPTLC) (Linomat V, Camag, Muttenz, Switzerland). Additionally, rat-specific tumor necrosis factor-α (TNF-α), Interleukin (IL)-1β, and IL-6 enzyme-linked immunosorbent assay (ELISA) kit were obtained from Bethyl Laboratories Inc. (Montgomery, TX, USA).

### QTN (3,5,7,3′,4′-Pentahydroxy flavone) isolation and characterization

Purification, isolation, and characterization of QTN (3,5,7,3′,4′-Pentahydroxy flavone) was performed according to a method reported elsewhere^14^. Briefly, air-dried powdered leaves of *Madhuca indica* J. F. Gmel. (500 g) was extracted with methanol (MI-ALC), and initial phytochemical screening was performed to identify various phytochemicals such as flavonoids, alkaloids, steroid & phenols, etc.^14^. Further fractionation of MI-ALC was performed with chloroform and mixed with acetone (70 mL) for column chromatography analysis. Elution was carried out with mobile condition phase (acetone: n-hexane (0.5:9.5)) at a flow rate of 2 mL/min. HPTLC analysis of various fractions (A-H) was performed with the following reagents:

- A stock solution of standard (quercetin) at 1000 µg/mL with a final concentration of 50 ng/µL
- Mobile phase of Toluene: Ethyl Acetate: Formic Acid (6: 3.5: 0.5 v/v/v)

The drug was observed to show a considerable absorbance at 370 nm.

Among all the fractions, active compound isolation was carried out in fraction D using preparative HPTLC. Furthermore, FT-IR and LC–MS spectroscopy were used to elucidate the chemical structure of the isolated compound.

### Adjuvant-induced polyarthritis (AIA)

FCA (0.1 mL, intradermal) was injected into the tail of the rats to induce AIA in female rats (150–180 g; 5 groups, i.e., group II to VI, n = 18 each group)^19^. Following the injection, 32 days were allowed for development of arthritis. A separate group of rats (group I, n = 18) was maintained as normal and did not receive FCA. After the development of AIA (32 days), animals received either dimethyl sulfoxide (DMSO) (0.5%, 10 mg/kg, p.o. in group I and II) or leflunomide (10 mg/kg in 0.5% DMSO, as a standard in group III) or QTN (5, 10 and 20 mg/kg in 0.5% DMSO in group IV to VI) for the next 28 days^14^. A solution of leflunomide or QTN was freshly prepared daily in 0.5% DMSO and administered to rats by oral gavage (1 mL) according to their dose based on body weight of rats.

Plethysmometer (UGO Basile Italy) was used to determine paw volume^20^. Pain latency against mechanical hyperalgesia (paw withdrawal threshold) was determined by using Randall-Selitto, i.e., paw pressure test (Ugo Basile Model 7200) as well as von Frey hair application^20^. Hargreaves apparatus (UGO Basile, SRL Biological Research Apparatus, Italy) was used to determine paw withdrawal latency^21,22^.

### Biochemical estimation

On the last day of study (day 60), rats were anesthetized with ether and retro-orbital plexus was used to withdraw blood. After blood withdrawal, rats were sacrificed by cervical dislocation. Immediately synovial tissues were isolated and stored at − 70 °C. Levels of ESR (Erythrocyte Sedimentation Rate), CRP (C-reactive protein), WBC (White blood cell), Hb (hemoglobin), RBC (Red blood cell), and PLT (platelets) were estimated in blood^20^, whereas turbidity, albumin, AST (aspartate transaminase), ALP (alkaline phosphatase), ALT (alanine transaminase), and TC (total cholesterol) were measured in serum using reagent kits (Accurex Biomedical Pvt. Ltd., Mumbai, India)^23^.

#### Determination of tissue oxido-nitrosative stress

Tris–HCl buffer (0.1 M, pH 7.4) was used to prepare homogenates of synovial tissue, and the supernatant was used to determine oxido-nitrosative stress (n = 5–6), i.e., MDA (lipid peroxidation), GSH (reduced glutathione), SOD (superoxide dismutase) and NO (nitric oxide) according to previously described method^24^.

#### Determination of synovial pro-inflammatory cytokines (TNF-α, IL-1β, and IL-6) levels

Pro-inflammatory cytokine levels, i.e., TNF-α, IL-1β, and IL-6 of synovial tissue (n = 5–6) were determined using a commercially available enzyme-linked immunosorbent assay (ELISA) kit according to the manufacturer’s protocol (Bethyl Laboratories Inc., Montgomery, TX, USA). TNF-α, IL-1β, and IL-6 were determined from a standard curve for the combination of these cytokines. The concentrations were expressed as pg/mg of protein. Briefly, the quantifications of TNF-α, IL-1β, and IL-6 were done in accordance to the protocol provided with Bethyl Laboratories Inc Rat TNF-α, IL-1β, and IL-6 immunoassay kit. Rat TNF-α, IL-1β, and IL-6 immunoassay was a 4.5 h solid-phase ELISA designed to measure rat TNF-α, IL-1β, and IL-6 levels. The assay employed a sandwich enzyme immunoassay principle. A monoclonal antibody specific to rat TNF-α, IL-1β, and IL-6 was pre-coated on the microplates. Standards, control, and samples were pipetted into the wells, and any rat TNF-α, IL-1β, and IL-6 present in the sample was thus bound by the immobilized antibody. After washing away the unbound substance, an enzyme-linked polyclonal antibody specific to rat TNF-α, IL-1β, and IL-6 was pipetted into the microtitre wells. Any unbound antibody was washed off, and then a substrate solution was added to the wells. The enzymatic reaction produced a blue product that turned yellow when the stop solution was added. The intensity of the color generated was measured, which was proportional to the amount of rat TNF-α, IL-1β, and IL-6 bound in the initial steps. A standard curve was run on each assay plate using recombinants of the TNF-α, IL-1β, and IL-6 in serial dilutions. The sample values were then read, and calculations were made according to the standard curve. Values were expressed as means ± SEM. The levels of TNF-α, IL-1β, and IL-6 were expressed as units per mL.

#### Determination of synovial protein expressions of COX-2, Ikβα, NF-kβ, and P2X7

The protein expressions of COX-2, Ikβα, NF-kβ, and P2X7 in synovial tissue (n = 4) were determined using Western blot analysis according to a method reported elsewhere^24^. Briefly, synovial tissue was sonicated in tissue protein extraction reagent (Thermo Fisher Scientific, Inc.). The lysates were centrifuged at 10,000 xg for 10 min at 4 °C. Protein concentration was determined using a Bicinchoninic Acid (BCA) assay kit (Beyotime Shanghai, China) on ice for 30 min. Equal amounts of extracted protein samples (50 μg) were separated by 10% SDS-PAGE (sodium dodecyl sulfate–polyacrylamide gel electrophoresis) and transferred onto polyvinylidene difluoride membranes. The membranes were blocked with 5% non-fat dry milk at 37 °C for 1 h and incubated overnight at 4 °C with the primary antibodies that recognized COX-2, Ikβα, NF-kβ, P2X7 and GAPDH. Anti-rabbit horseradish-linked IgG was used as the secondary antibody, which was incubated at 37 °C for 2 h. Protein bands were visualized using Chemiluminescent kit (Bio-Rad Laboratories, Inc.) and GAPDH served as the loading control.

#### Histopathology of tibiotarsal joint

Ankle joints from three rats of each group were separated, cleaned, washed in cold physiological saline, and preserved in 10% formaldehyde solution until histopathological studies. At the time of staining, sections of tibiotarsal joints were cut (5 μm thickness) with the help of microtome, deparaffinated, and stained using hematoxylin and eosin (H and E) stain. The specimens were mounted on slides using Distrene Phthalate Xylene (DPX). Sections were examined under a light microscope (Olympus DP71, DP-BSW Ver.03.03, Olympus Medical Systems India Private Limited, India) to obtain a general impression of the histopathological features of the specimen and infiltration of cells in epithelium and sub-epithelium. The intensity of histological aberrations in the tibiotarsal joint was graded as Grade 0 (not present)l; Grade 1 (slight/minimal); Grade 2 (mild); Grade 3 (moderate) or Grade 4 (severe) as described in the literature^25^.

### Statistical analysis

GraphPad Prism 5.0 software (GraphPad, San Diego, CA) was used to perform data analysis. Data are expressed as mean ± standard error mean (SEM) and analyzed using One-Way ANOVA followed by Tukey’s multiple range post hoc analysis (for parametric tests including body weight, joint diameter, paw volume, paw withdrawal latency, paw withdrawal threshold, serum AST, ALT, ALP, albumin, CRP, rheumatoid factor, hematological parameters, ESR, antioxidant parameters, cytokines levels and protein expressions (COX-2, Ikβα, NF-kβ, and P2X7) or Kruskal–Wallis test for post hoc analysis (non-parametric tests, i.e., histopathology score). A value of *p* < 0.05 was considered to be statistically significant.

### Ethical approval

The research protocol (no. XCH202013771) was approved by the Institutional animal ethics committee (IAEC) of Xi’an Central Hospital. Guidelines outlined in the Guide for the Care and Use of Laboratory Animals of the National Institutes of Health and the ARRIVE (Animal Research: Reporting of *In-vivo* Experiments) guidelines (http://www.nc3rs.org/ARRIVE) were followed to perform all the experiments.

## Results

### Compound D3 Isolation and characterization

In the yield of MI-ALC, its fractions D and D 3 were 26.8%, namely 5.42 g, and 30.8 mg. HPTLC linearity profile of marker compound (quercetin) and QTN from 50 to 500 ng/µl at 370 nm are shown in Supplementary File 1A and B. The results of regression analysis of the calibration curves showing the correlation coefficient (0.980), intercept (− 550.1), and slope (40.50) are given in Supplementary File 1C. The UV spectrum of the test sample is superimposable with that of standard quercetin, indicating the purity of the peak (Supplementary File 1D).

**Figure 1:**
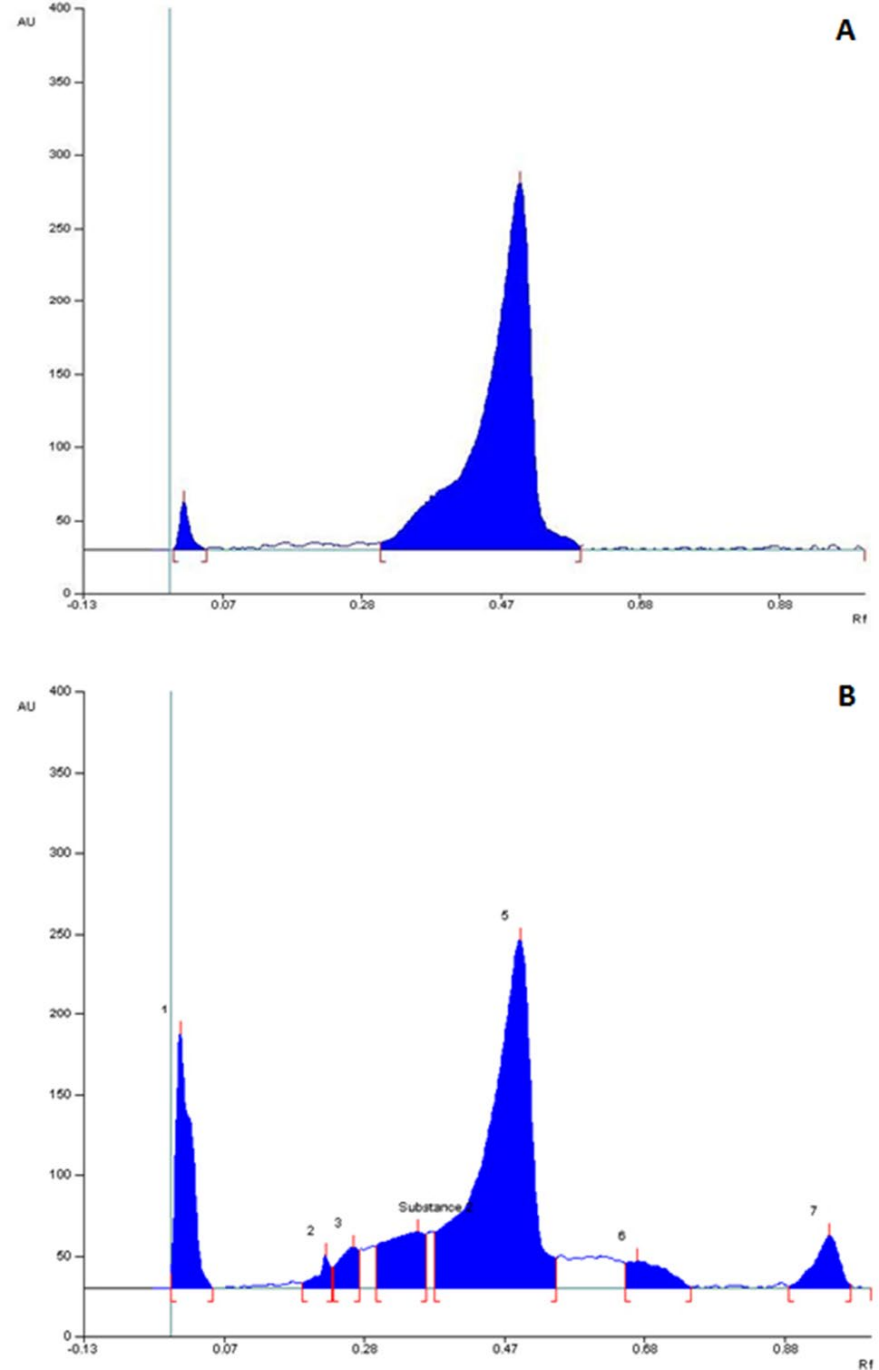
HPTLC profile of quercetin (**A**) and HPTLC profile of chloroform fraction D (QTN) of MI-ALC **(B).** Eluent–toluene: ethyl acetate: formic acid (6: 3.5: 0.5) (v/v/v). The detection was at 370 nm.

Figure 1A depicts chromatograph of marker compound (i.e., quercetin), and Fig. 1B depicts chromatograph of isolated fraction QTN. QTN is an amorphous powder with a melting point of 307–309 °C. FTIR spectrum of compound QTN is showed in Supplementary File 1E. The QTN showed a molecular ion peak at m/z 302 in the LC–MS spectrum, suggesting molecular formula C_15_H_10_O_7_ (Supplementary File 1F) and 3,5,7,3′,4′-Pentahydroxy flavone as the chemical structure which corroborated reported literature (Supplementary File 1F)^14^.

### Body weight, paw volume, joint diameter, paw withdrawal threshold, and latency

**Figure 2:**
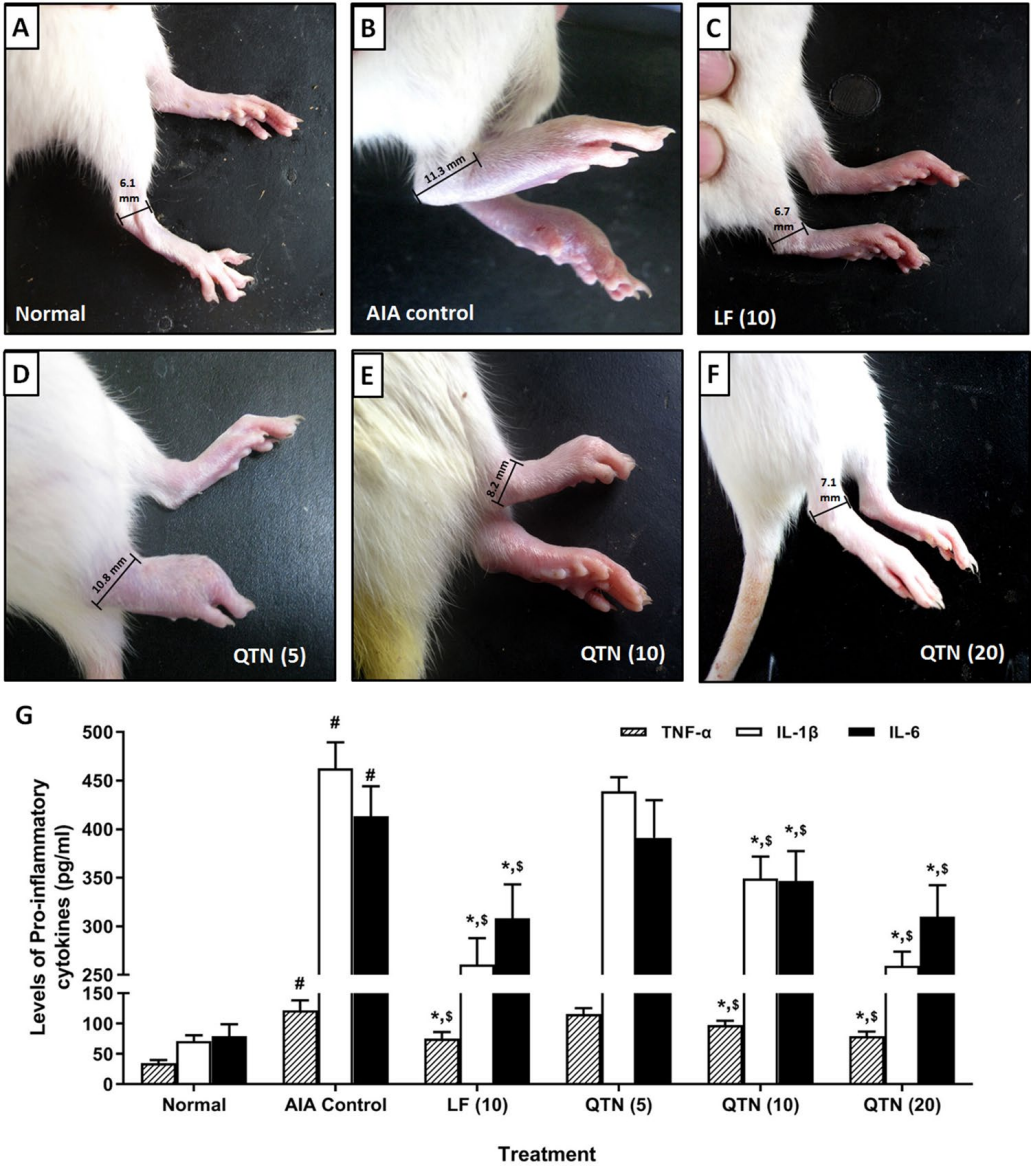
Effects of QTN on severity of ankle inflammation in rats **(A-F).** Representative images of paw from (**A**) normal rats, (**B**) AIA control rats with higher degree of inflammation, (**C**) Leflunomide (10 mg/kg) treated rats with reduced ankle inflammation, (D) QTN (5 mg/kg) treated rats with severe inflammation, (**E**) QTN (10 mg/kg) and (**F**) QTN (20 mg/kg) treated rats with reduced ankle inflammation. Effects of QTN on FCA-induced alterations in pro-inflammatory cytokines levels in synovial tissues **(G).** Values in parentheses indicate a dose in mg/kg (n = 6). Data was analyzed by one-way ANOVA followed by Tukey’s multiple comparisons test. For comparison with AIA-control group: *p < 0.05, comparison with normal group: ^#^*p* < 0.05 and comparison with one another: ^$^*p* < 0.05. *AIA* adjuvant-induced arthritis, *LF* leflunomide, *QTN* 3,5,7,3′,4′-pentahydroxy flavone, *TNF-α* tumor necrosis factor-alpha, *ILs* interleukins.

Body weight markedly decreased (*p* < 0.05), whereas joint diameter, paw volume, paw withdrawal threshold, and latency increased significantly (*p* < 0.05) in AIA control rats compared to normal rats. Treatment with leflunomide (10 mg/kg) effectively inhibited (*p* < 0.05) FCA-induced alterations in body weight, paw volume, joint diameter, paw withdrawal threshold, and latency compared to AIA control rats. QTN (10 and 20 mg/kg) treatment predominately increased (*p* < 0.05) body weight and significantly decreased (*p* < 0.05) paw volume, joint diameter, paw withdrawal threshold, and latency when compared with AIA control rats. (Table 1 and Fig. 2).

**Table 1:** Effects of QTN on FCA-induced alterations in body weight, change in joint diameter, change in paw volume, paw withdrawal latency, and paw withdrawal threshold (Von-Frey hair and paw pressure test).

Treatment	AUC (Body weight (gm))	AUC (change in joint diameter (mm))	AUC (change in Paw volume (mL))	AUC (Paw withdrawal latency (sec))	AUC (Paw withdrawal threshold (g))	AUC (Paw withdrawal threshold (g))
Normal	6299.00 ± 56.77	0.00 ± 0.00	0.18 ± 0.05	242.50 ± 6.92	1803.00 ± 41.74	8315.00 ± 72.92
AIA Control	5468.00 ± 37.06^#^	87.06 ± 0.44^#^	91.84 ± 1.62^#^	120.60 ± 7.28^#^	791.70 ± 24.85^#^	4884.00 ± 130.20^#^
LF (10)	5773.00 ± 96.24*^,$^	71.37 ± 0.99*^,$^	70.64 ± 0.84*^,$^	154.60 ± 7.94*^,$^	1037.00 ± 23.53*^,$^	5978.00 ± 160.60*^,$^
QTN (5)	5552.00 ± 103.6	86.18 ± 1.02	90.72 ± 0.58	127.80 ± 8.55	832.60 ± 12.94	5110.00 ± 106.30
QTN (10)	5681.00 ± 75.02*^,$^	79.64 ± 1.06*^,$^	81.50 ± 0.62*^,$^	142.90 ± 9.78*^,$^	897.20 ± 18.42*^,$^	5503.00 ± 45.96*^,$^
QTN (20)	5771.00 ± 72.76*^,$^	71.74 ± 0.85*^,$^	70.33 ± 0.96*^,$^	156.40 ± 8.66*^,$^	1034.00 ± 26.61*^,$^	5924.00 ± 91.91*^,$^

Values in parentheses indicate a dose in mg/kg (n = 6). Data were analyzed by one-way ANOVA followed by Tukey’s multiple comparisons test. For comparison with AIA-control group: *p < 0.05, comparison with normal group: ^#^*p* < 0.05 and comparison with one another: ^$^*p* < 0.05. *AIA* adjuvant-induced arthritis, *LF* leflunomide, *QTN* 3,5,7,3′,4′-pentahydroxy flavone, *AUC* area under curve.

### AST, ALT, ALP, albumin, CRP, and rheumatoid factor

When compared to normal rats, serum ALT, AST, ALP, and CRP markedly (*p* < 0.05) increased, whereas serum albumin level significantly (*p* < 0.05) decreased in the AIA control rats. Leflunomide (10 mg/kg) treatment effectively (p < 0.05) increased albumin level in serum whereas it markedly (*p* < 0.05) reduced levels of ALT, AST, ALP and CRP in serum compared to AIA control rats. The FCA-induced alterations in serum ALT, AST, ALP, CRP, and albumin levels were significantly (*p* < 0.05) attenuated by QTN (10 and 20 mg/kg) when compared with AIA control. These alterations in levels of serum AST, ALT, ALP, albumin, and CRP were more effectively (*p* < 0.05) attenuated by leflunomide (10 mg/kg) when compared to QTN treatment. (Table 2).

**Table 2:** Effects of QTN on FCA-induced alterations in serum AST, ALT, ALP, albumin, CRP, and rheumatoid factor levels.

Treatment	AST (U/mL)	ALT (U/mL)	Alkaline phosphatase (U/L)	Albumin (g/dl)	CRP (mg/L)	Rheumatoid factor (IU/mL)
Normal	39.95 ± 3.02	33.78 ± 1.83	69.24 ± 5.42	6.52 ± 0.38	0.99 ± 0.04	0.00 ± 0.00
AIA Control	122.90 ± 2.84^#^	165.80 ± 7.45^#^	435.00 ± 11.81^#^	2.00 ± 0.22^#^	7.65 ± 0.34^#^	60.25 ± 1.55^#^
LF (10)	59.56 ± 3.74*^,$^	51.11 ± 5.14*^,$^	136.7 ± 5.59*^,$^	6.25 ± 0.31*^,$^	2.60 ± 0.24*^,$^	38.58 ± 1.47*^,$^
QTN (5)	118.70 ± 4.26	146.60 ± 3.80	423.20 ± 6.50	2.25 ± 0.26	7.02 ± 0.18	56.96 ± 1.61
QTN (10)	88.02 ± 3.71*^,$^	90.45 ± 3.86*^,$^	276.40 ± 12.82*^,$^	4.05 ± 0.21*^,$^	4.74 ± 0.27*^,$^	47.86 ± 1.74*^,$^
QTN (20)	62.75 ± 1.42*^,$^	52.28 ± 3.82*^,$^	139.30 ± 7.38*^,$^	5.78 ± 0.22*^,$^	2.67 ± 0.13*^,$^	38.88 ± 0.97*^,$^

Values in parentheses indicate a dose in mg/kg (n = 6). Data were analyzed by one-way ANOVA followed by Tukey’s multiple comparisons test. For comparison with AIA-control group: *p < 0.05, comparison with normal group: ^#^*p* < 0.05 and comparison with one another: ^$^*p* < 0.05. *AIA* adjuvant-induced arthritis, *LF* leflunomide, *QTN* 3,5,7,3′,4′-pentahydroxy flavone, *AST* aspartate aminotransferase, *ALT* alanine transaminase, *CRP* c-reactive protein.

The subplantar administration of FCA resulted in a marked increase (p < 0.05) in the rheumatoid factor in AIA control rats compared to normal rats. As compared to AIA control rats, leflunomide (10 mg/kg) and QTN (10 and 20 mg/kg) treatment significant decreased (*p* < 0.05) rheumatoid factor. (Table 2).

### Hematological parameters and ESR levels

A significant decrease (*p* < 0.05) in RBCs and Hb levels, whereas a marked increase (*p* < 0.05) in WBCs, platelet, and ESR levels were observed in AIA control rats when compared with normal rats. Treatment with Leflunomide (10 mg/kg) effectively ameliorated (*p* < 0.05) hematological and ESR variations when compared with AIA control rats. QTN (10 and 20 mg/kg) significantly increased (*p* < 0.05) levels of RBCs and Hb whereas effectively reduced (*p* < 0.05) WBCs, platelet and ESR levels when compared with AIA control rats. However, leflunomide (10 mg/kg) more significantly (*p* < 0.05) attenuated FCA-induced hematological and ESR variations as compared to QTN treatment. (Table 3).

**Table 3:** Effects of QTN on FCA-induced alterations in hematological parameters and ESR level.

Treatment	RBC (X 10^6^/µL)	WBC (X 10^3^/µL)	Hb (g/dL)	Platelets (X 10^9^/L)	ESR (mm)
Normal	5.55 ± 0.23	5.78 ± 0.30	15.83 ± 0.52	912.80 ± 40.47	3.64 ± 0.23
AIA Control	1.95 ± 0.17^#^	12.45 ± 0.41^#^	9.81 ± 0.55^#^	1658.00 ± 35.94^#^	14.69 ± 0.27^#^
LF (10)	4.91 ± 0.26*^,$^	6.84 ± 0.29*^,$^	15.41 ± 0.73*^,$^	1013.00 ± 37.21*^,$^	9.97 ± 0.44*^,$^
QTN (5)	2.02 ± 0.21	11.92 ± 0.25	10.10 ± 0.43	1633.00 ± 38.45	14.30 ± 0.44
QTN (10)	2.94 ± 0.24*^,$^	9.55 ± 0.25*^,$^	12.16 ± 0.60*^,$^	1453.00 ± 37.97*^,$^	12.63 ± 0.42*^,$^
QTN (20)	4.45 ± 0.20*^,$^	6.96 ± 0.28*^,$^	15.39 ± 0.33*^,$^	1048.00 ± 27.22*^,$^	10.19 ± 0.43*^,$^

Values in parentheses indicate a dose in mg/kg (n = 6). Data were analyzed by one-way ANOVA followed by Tukey’s multiple comparisons test. For comparison with AIA-control group: *p < 0.05, comparison with normal group: ^#^*p* < 0.05 and comparison with one another: ^$^*p* < 0.05. *AIA* adjuvant-induced arthritis, *LF* leflunomide, *QTN* 3,5,7,3′,4′-pentahydroxy flavone, *RBC* red blood cells, *WBC* white blood cells, *ESR* erythrocyte sedimentation rate.

### Synovial oxido-nitrosative stress

Synovial GSH and SOD markedly decreased (*p* < 0.05), whereas NO and MDA levels effectively increased (*p* < 0.05) in AIA control rats when compared with normal rats. Leflunomide (10 mg/kg) markedly increased (*p* < 0.05) synovial GSH and SOD levels, whereas NO and MDA levels decreased effectively (*p* < 0.05) in synovial fluid when compared with AIA control rats. Similarly, QTN (10 and 20 mg/kg) effectively attenuated (*p* < 0.05) FCA-induced increased synovial oxido-nitrosative stress when compared with AIA control rats. However, elevated MDA and decreased GSH levels were more effectively (*p* < 0.05) attenuated by QTN (20 mg/kg) when compared with leflunomide (10 mg/kg). (Table 4).

**Table 4:** Effects of QTN on FCA-induced alterations in antioxidant parameters in synovial tissues.

Treatment	SOD (U/mg of protein)	GSH (µg/mg of protein)	MDA (nM/mg of protein)	NO (μg/mL)
Normal	5.75 ± 0.27	81.76 ± 2.57	0.97 ± 0.11	116.50 ± 13.63
AIA Control	3.24 ± 0.26^#^	52.20 ± 3.35^#^	2.46 ± 0.09^#^	519.00 ± 11.21^#^
LF (10)	4.55 ± 0.25*^,$^	68.51 ± 2.09*^,$^	1.67 ± 0.13*^,$^	168.80 ± 14.86*^,$^
QTN (5)	3.31 ± 0.24	55.80 ± 1.67	2.39 ± 0.09	486.10 ± 13.86
QTN (10)	3.99 ± 0.27*^,$^	63.13 ± 2.21*^,$^	1.95 ± 0.12*^,$^	400.40 ± 13.97*^,$^
QTN (20)	4.55 ± 0.24*^,$^	69.32 ± 1.52*^,$^	1.62 ± 0.13*^,$^	284.00 ± 14.63*^,$^

Values in parentheses indicate a dose in mg/kg (n = 5–6). Data were analyzed by one-way ANOVA followed by Tukey’s multiple comparisons test. For comparison with AIA-control group: *p < 0.05, comparison with normal group: ^#^*p* < 0.05 and comparison with one another: ^$^*p* < 0.05. *AIA* adjuvant-induced arthritis, *LF* leflunomide, *QTN* 3,5,7,3′,4′-pentahydroxy flavone, *SOD* superoxide dismutase, *GSH* glutathione peroxidase, *MDA* malondialdehyde, *NO* Nitric oxide.

### Synovial ILs (IL-1β, and IL-6) and TNF-α protein levels

Intraperitoneal administration of FCA resulted in a significant increase (*p* < 0.05) in ILs (IL-1β, and IL-6) and TNF-α protein levels in the synovial tissue of AIA control rats when compared with normal rats. Leflunomide (10 mg/kg) markedly inhibited (*p* < 0.05) FCA-induced increased synovial ILs (IL-1β, and IL-6) and TNF-α protein levels as compared to AIA control rats. QTN (10 and 20 mg/kg) also effectively decreased (*p* < 0.05) elevated synovial ILs (IL-1β, and IL-6) and TNF-α protein levels when compared with AIA control rats. The elevated synovial TNF-α and IL-6 protein levels were more effectively (*p* < 0.05) attenuated by leflunomide (10 mg/kg) when compared with QTN. (Table 4).

### Synovial COX-2, Ikβα, NF-kβ, and P2X7 protein expressions

**Figure 3:**
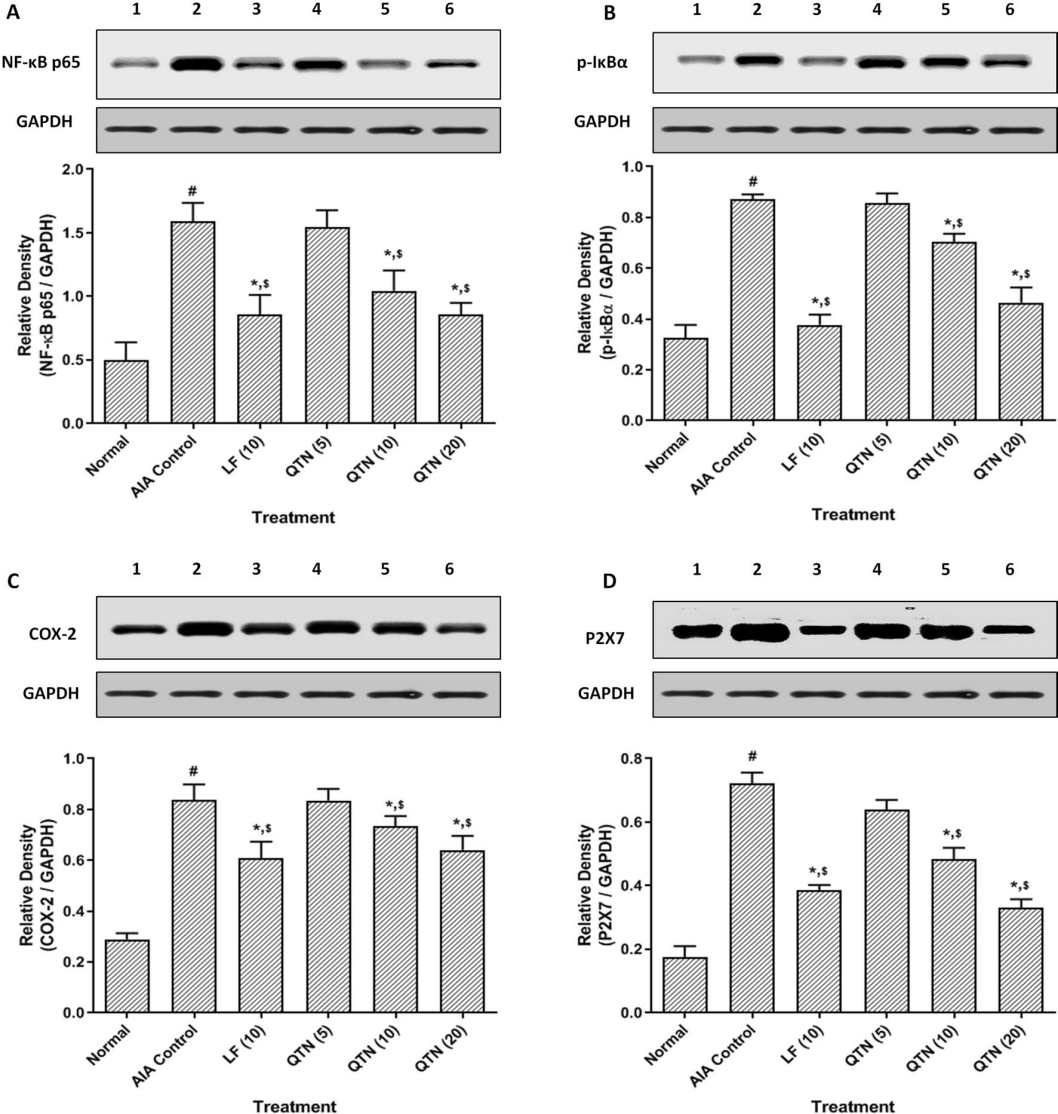
Effects of QTN on FCA-induced alterations in protein expression of NF-kβ p65 **(A)**, p-Ikβα (**B**), COX-2 (**C**), and P2X7 (**D**) in synovial tissues. Data are expressed as mean ± SEM (n = 4) and analyzed by one-way ANOVA followed by Tukey’s multiple range test. For comparison with AIA-control group: *p < 0.05, comparison with normal group: ^#^*p* < 0.05 and comparison with one another: ^$^*p* < 0.05. Normal group protein expression (lane 1); AIA control group protein expression (lane 2); Leflunomide group protein expression (lane 3) and QTN (5, 10, and 20 mg/kg) group protein expression (lane 4–6). *AIA* adjuvant-induced arthritis, *LF* leflunomide, *QTN* 3,5,7,3′,4′-pentahydroxy flavone, *NF-kβ* nuclear factor kappa beta, *Ikβα* nuclear factor of kappa light polypeptide gene enhancer in B cells inhibitor-α, *COX-2* cyclooxygenase-2, *P2X7* ATP-activated P2 purinergic receptors.

The protein expressions of COX-2, Ikβα, NF-kβ, and P2X7 in synovial tissues of AIA control rats was up-regulated markedly (*p* < 0.05) after administration of FCA when compared with normal rats. Leflunomide (10 mg/kg) effectively (*p* < 0.05) down-regulated protein expressions of COX-2, Ikβα, NF-kβ, and P2X7 in synovial tissues when compared to AIA control rats. Moreover, QTN (10 and 20 mg/kg) also effectively (*p* < 0.05) inhibited FCA-induced up-regulated protein expressions of COX-2, Ikβα, NF-kβ, and P2X7 in synovial tissues when compared with AIA control rats. QTN (20 mg/kg) showed more effective (*p* < 0.05) down-regulation in the protein expression of P2X7 in synovial tissues as compared to leflunomide (10 mg/kg). (Fig. 3 and Supplementary File 1G-K).

### Histopathology of the tibiotarsal joint

**Figure 4:**
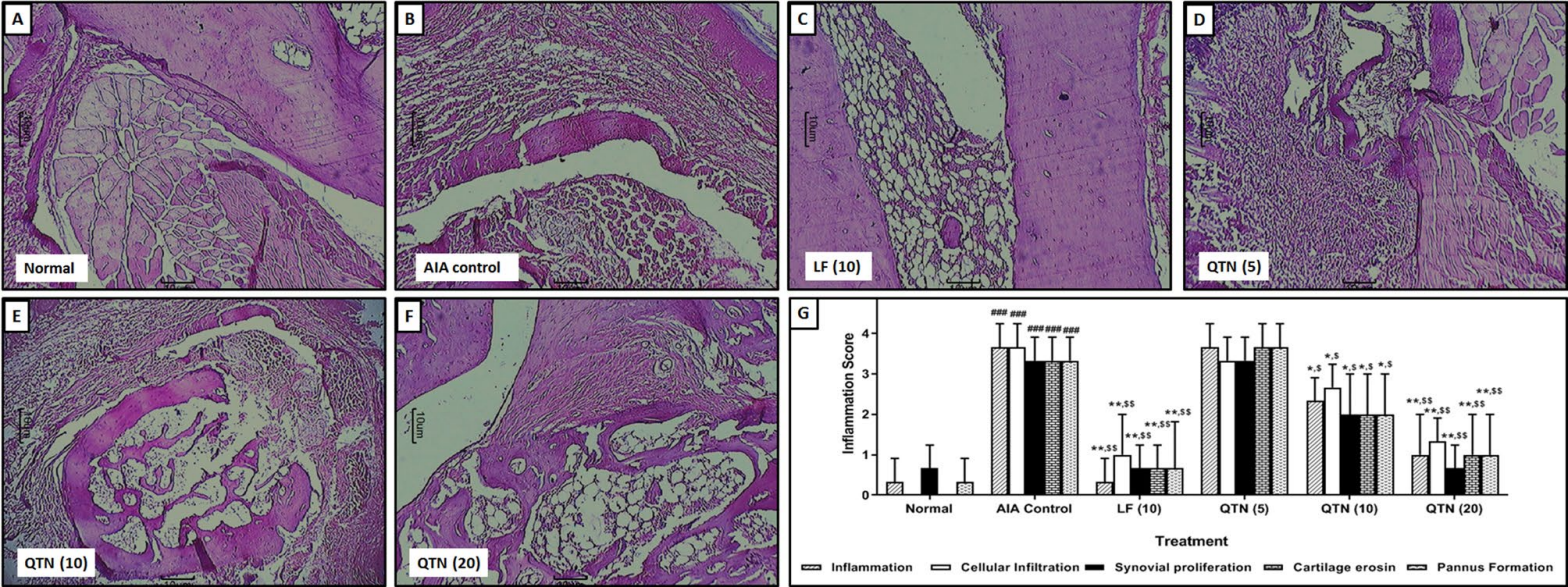
Effects of QTN on the histopathology of tibiotarsal joints. Representative histological images from (**A**) normal, (**B**) AIA control, (**C**) leflunomide (10 mg/kg), (**D**) QTN (5 mg/kg), (**E**) QTN (10 mg/kg) and (**D**) QTN (20 mg/kg) treated rats. Images stained with H&E (× 100). The quantitative representation of histological score (**G**). Data were expressed as mean ± S.E.M. (n = 3), and one-way ANOVA followed by the Kruskal–Wallis test was applied for post hoc analysis. For comparison with AIA-control group: *p < 0.05, comparison with normal group: ^#^*p* < 0.05 and comparison with one another: ^$^*p* < 0.05.

FCA administration resulted in marked histological aberrations in the tibiotarsal joint of AIA control rats reflected by an effective increase (*p* < 0.05) in inflammatory infiltration, synovial proliferation, cartilage erosion, and pannus formation (Fig. 4B) when compared with normal rats. Whereas, tibiotarsal joint of normal rats showed minimal inflammatory infiltration, synovial proliferation, and pannus formation (Fig. 4A). Leflunomide (10 mg/kg) treatment effective inhibited (*p* < 0.05) FCA-induced histological aberrations in tibiotarsal joint (Fig. 4C) when compared with AIA control rats. However, compared with AIA control rats, QTN (5 mg/kg) failed to produce any marked protection against histological aberrations induced by FCA in the tibiotarsal joint (Fig. 4D). But QTN (10 and 20 mg/kg) effectively decreased (*p* < 0.05) inflammatory infiltration, synovial proliferation, and pannus formation (Fig. 4E,F) when compared with AIA control rats. (Fig. 4G).

## Discussion

*Madhuca indica* is a traditional medicine rich in various phytoconstituents dominant with the presence of flavonoids. It has been widely used to manage various inflammatory disorders due to its inhibitory potential against histamine, serotonin, prostaglandin, and COX-2^16^. In the present study, we have assessed the anti-arthritic effect of an isolated phytoconstituent (3,5,7,3′,4′-Pentahydroxy flavone, i.e., QTN) from *Madhuca indica* Leaves on female Wistar rats after subplantar administration of FCA. The findings of the present investigation suggested that methanolic extract from *Madhuca indica* leaves exhibit anti-arthritic activity and its phytoconstituent 3,5,7,3′,4′-Pentahydroxy flavone (i.e., QTN) is responsible for its anti-arthritic potential. Furthermore, QTN exerts its anti-arthritic potential via attenuation of pro-inflammatory cytokines (TNF-α*,* IL-1β, and IL-6), oxido-nitrosative stress, and NF-kβ, Ikβα, COX-2, and P2X7 expressions. Results of present investigation are in line with the findings of previous investigators where administration of Pentahydroxy flavone, i.e., quercetin ameliorated collagen-induced arthritis^26^ and adjuvant arthritis^27^ in experimental animal models.

Previous studies showed that elevated paw thickness is an evidence of arthritis induction^2,7,28,29^. Determination of paw thickness using a plethysmometer is a well-established and standardized method during AIA-induced arthritis^2,7,29^. In the present study, the inflamed rat’s edematous hind paw was estimated using a plethysmometer. It was further subjected to a constant force to assess the pain threshold that was examined by the Randall-Selitto assay method. Treatment with QTN significantly decreased paw thickness, which might be due to inflammatory mediator inhibition suggesting its anti-inflammatory property against FCA-induced arthritis. This potential of cytokine blockage in pain nervous fibers by QTN might be responsible for increased pain threshold testifying its analgesic effect. The presence of flavonoid moiety in the methanolic extract of *Madhuca indica* could be responsible for its anti-inflammatory, analgesic, and anti-nociceptive activities. The findings of the current study corroborate the results of earlier investigators where *Madhuca indica* phytoconstituents showed anti-inflammatory potential via reduction of TNF-α and IL-1β^13^.

Oxidative stress plays a central role in the activation and maintenance of painful arthritis^30^. It has been reported that increased production of ROS (reactive oxygen species) such as hydroxyl, hydrogen peroxide, and superoxide radicals contribute to elevated oxidative stress^4,7,20^. This elevated oxidative stress further decreased protective antioxidant moieties (GSH and SOD) that caused elevation of MDA (lipid peroxidation), thus damaging biomembrane macromolecules^22,31,32^. The FCA administration caused a marked reduction in synovial GSH and SOD, whereas the MDA level increased effectively. However, treatment with QTN significantly attenuated FCA-induced decreased GSH and SOD in the synovial tissue suggesting its antioxidant potential that might support its anti-arthritic mechanism. These findings are in accordance with the results of an earlier study where QTN isolated from *Madhuca indica* was shown to exert its potential via attenuation of oxidative stress^14^.

Numerous researchers have suggested that the Nuclear Factor kappa-light-chain-enhancer of activated B cells (NF-kB) plays a vital role in the induction and maintenance of immune-inflammatory disease modulation of various biomolecules such as COX-2 and pro-inflammatory cytokines^7,33^. During the resting state, the NF-kB remains unstimulated and retains an inactive state in the cytoplasm. Whereas, IκB kinase, which is an enzyme complex, plays an essential role in the upstream NF-κB signal transduction pathway, and its phosphorylated activation leads to subsequent ubiquitination and degradation of 26S proteasome^34^. This cascade leads to NF-kB translocation to the nucleus from the cytoplasm, where it modulates the expression of various genes, including pro-inflammatory cytokines^34^. In the present investigation, activated expressions of IκBα and NF-kB were found to be significantly up-regulated in synovial of AIA control rats after administration of FCA whereas QTN treatment significantly down-regulated these expressions of pro-inflammatory cytokines via attenuation of IκBα phosphorylation and thus inactivation of NF-kB.

Purinergic Receptor-X 7 (P2X7), a protein-coding gene from the purinoceptors family, has been suggested to play a central role in the induction and maintenance of an array of diseases associated with bone cartilage such as RA^35^. It is known to play a vital role in bone remodeling via activation of various mediators, including IL-1β and prostaglandins, in the synovial fluid^35^. It has been suggested that activation of ectonucleotidases degrade extracellular ATP, which results in the formation of active molecules such as adenosine or pyrophosphates^36^. These active molecules further promote the activation of alternative macrophages and thus initiate the release of pro-inflammatory signaling^36^. Therefore, extracellular metabolism of ATP by P2X7 modulates the sequence of inflammatory influx and thus initiates the pathogenesis of RA^35^. In this view, inhibition of P2X7 receptor activation would be beneficial for the management of RA. In the current investigation, treatment with QTN significantly inhibits P2X7 activation, which might reduce the bone and cartilage damage in RA.

Recently an array of isolated phytoconstituents from herbal origin have been implicated in the management of arthritis clinically. Studies have investigated the potential of various moieties such as Pycnogenol® from *Pinus pinaster* Aiton, Curcuminoids from *Curcuma longa*, Bromelain from *Ananas comosus,* etc. for the symptomatic relief of RA^37^. Furthermore, a researcher suggested that a moiety bearing carbonyl group at C-4 and a hydroxyl group at C-3 or C-5 in their structure have chelation ability with metal ions, helpful in exerting its antioxidant property^24^. In the current study, an isolated moiety from methanolic extract of leaves of *Madhuca indica,* i.e., QTN (3,5,7,3′,4′-Pentahydroxy flavone) also bears such hydroxyl and carbonyl groups in its structure, indicating promising antioxidant potential. Thus, QTN can be considered as a potential therapeutic moiety for the management of RA clinically.

## Conclusion

A phytoconstituent isolated from methanolic extract *Madhuca indica* leaves, identified as 3,5,7,3′,4′-Pentahydroxy flavone (i.e., QTN), exhibits anti-arthritic activity. QTN ameliorates FCA-induced hyperalgesia through attenuation of elevated inflammatory mediators (NF-kβ, Ikβα, COX-2, and P2X7), oxido-nitrosative stress, and pro-inflammatory cytokines (ILs and TNF-α) experimental rats.

## Supplementary Information

Supplementary Legends.

Supplementary Information.

## Data Availability

The datasets analyzed during the current study are available from the corresponding author on reasonable request.

## Abbreviations

AIA: Adjuvant induced arthritis
ALT: Alanine transaminase
ALP: Alkaline phosphatase
AST: Aspartate aminotransferase
CRP: C-reactive protein
COX-2: Cyclooxygenase-2
ESR: Erythrocyte sedimentation rate
FCA: Freund’s complete adjuvant
GSH: Reduced glutathione
ILs: Interleukins
LF: Leflunomide
LDL: Low-density lipoprotein
MDA: Malondialdehyde
NO: Nitric oxide
NF-kB: Nuclear factor-kappa beta
Ikβα: Nuclear factor of kappa light polypeptide gene enhancer in B cells inhibitor-α
ROS: Reactive oxygen species
SOD: Superoxide dismutase
TNF-α: Tumor necrosis factor-alpha
P2X7: ATP-activated P2 purinergic receptors
QTN: 3,5,7,3′,4′-Pentahydroxy flavone
