# Supplementary material for: Pentahydroxy flavonoid isolated from Madhuca indica ameliorated adjuvant-induced arthritis via modulation of inflammatory pathways
Source: Sci Rep. 2021 Sep 9;11:17971. doi: 10.1038/s41598-021-97474-2 (PMC8429448; doi:10.1038/s41598-021-97474-2)
Supplement: Supplementary file 1 — Supplementary Legends. [file 41598_2021_97474_MOESM1_ESM.doc]

# Supplementary File

**Supplementary File 1:** HPTLC linearity profile of marker compound (quercetin) from 50 to 500 ng/µl at 370 nm (A), HPTLC linearity profile of QTN from 50 to 500 ng/µl at 370 nm (B), Calibration curve plot for QTN (C) and Super imposable spectra of marker compound (quercetin) and QTN (D). FT-IR spectra of D3 (3,5,7,3′,4′- Pentahydroxy flavone) (E) and LC-MS spectra of D3 (3,5,7,3′,4′- Pentahydroxy flavone) with its structure (F). Full scans of Western blot data (G-K).
