# Supplementary figures and images for: Pentahydroxy flavonoid isolated from Madhuca indica ameliorated adjuvant-induced arthritis via modulation of inflammatory pathways
Source: Sci Rep. 2021 Sep 9;11:17971. doi: 10.1038/s41598-021-97474-2 (PMC8429448; doi:10.1038/s41598-021-97474-2)

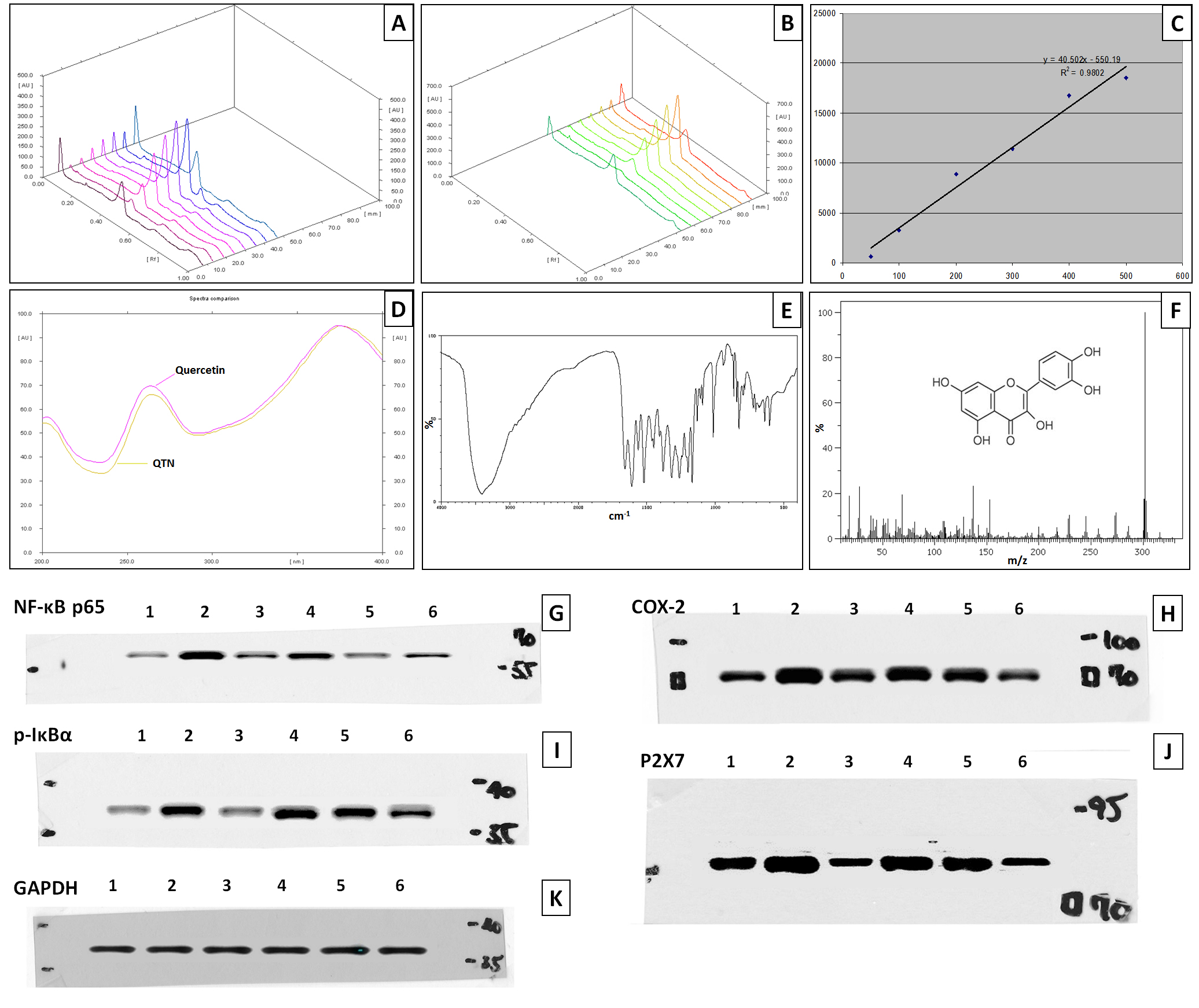

Supplement: Supplementary file 2 — Supplementary Information. [file 41598_2021_97474_MOESM2_ESM.jpg]
